# Validation of the Greek version of Mother's Autonomy in Decision Making (MADM) scale

**DOI:** 10.18332/ejm/189495

**Published:** 2024-07-08

**Authors:** Eleni Serpetini, Antigoni Sarantaki, Aikaterini Lykeridou, Sofia Tzamaria, Athina Diamanti

**Affiliations:** 1 Faculty of Health and Caring Sciences, Department of Midwifery, University of West Attica, Athens, Greece

**Keywords:** MADM, midwifery, pregnancy, home birth, maternal care, autonomy

## Abstract

**INTRODUCTION:**

Ensuring expectant mothers have the capacity to make well-informed decisions regarding their prenatal care, encompassing medical interventions, and birthing preferences are crucial for fostering favorable health outcomes for both mother and newborn. The Mother's Autonomy in Decision Making (MADM) scale serves as a commonly utilized tool for evaluating the autonomy of pregnant women in the decision-making processes related to prenatal care and childbirth. The aim of this study is to validate the MADM scale in women who had at least one home childbirth experience in Greece.

**METHODS:**

A retrospective online survey collected data from Greek women with home childbirth experience (January 2010 – December 2023). We utilized a self-administered questionnaire and the Greek version of the MADM scale.

**RESULTS:**

The study included 162 women, predominantly of Greek nationality (94.4%) and residing in Attica (54%). The MADM scale showed a median score of 38. The confirmatory factor analysis indicated acceptable fit and reliability (comparative fit index, CFI=0.92; Tucker-Lewis index, TLI=0.91; root mean square error of approximation, RMSEA=0.07; Cronbach's α=0.92). Age correlated weakly negatively with the MADM scale score (Spearman’s rho= -0.166, p=0.035). Additionally, women attending antenatal preparation courses with a midwife before their first home birth had higher MADM scores (median 39 vs 35, p=0.037).

**CONCLUSIONS:**

The study underscores the importance of the MADM scale, demonstrating its reliability and validity for women living in Greece. Younger age and attending antenatal preparation courses with a midwife were associated with higher MADM scores, highlighting education's role in maternal autonomy.

## INTRODUCTION

In the realm of maternal healthcare, the autonomy of pregnant women in decision-making processes is a critical aspect that influences not only their health outcomes but also the well-being of their offspring^1^. The ability of expectant mothers to make informed choices about their prenatal care, including medical interventions and childbirth options, is essential for promoting positive maternal and neonatal health outcomes^2^. Different cultural contexts have led to the development of various measurement tools to assess and understand the autonomy of pregnant women in decision-making^3^.

Pregnant women use the Mother’s Autonomy in Decision Making (MADM) scale, a widely used instrument, to assess their autonomy in prenatal care and childbirth decision-making processes. Originally developed by Vedam et al.^4^, the MADM scale comprises multiple items designed to measure maternal autonomy, including decision-making ability, information-seeking behavior, and participation in healthcare discussions.

The cultural nuances and sociopolitical factors inherent in each context necessitate the validation of the MADM scale in specific populations to ensure its validity and reliability. Diverse cultural settings have validated and utilized the MADM scale, demonstrating its utility and applicability across different populations^5-9^.

Greece, with its unique cultural, social, and healthcare landscape, presents distinct challenges and opportunities for promoting maternal autonomy during pregnancy^10^. The availability of culturally sensitive and valid instruments to measure maternal autonomy in decision-making remains limited, particularly in the Greek context.

In Greece, the public healthcare system primarily organizes maternity care, with additional private options available for those who can afford them. Hospitals and maternity clinics provide maternity care in the public sector, offering services from prenatal care to delivery and postnatal support. Obstetricians and midwives typically play key roles in providing care throughout the pregnancy and childbirth process. While home birth is legal in Greece, it is not as common as hospital births. The majority of Greek women choose to give birth in hospitals or maternity clinics, where they can access medical professionals and the necessary equipment for safe deliveries. However, a small percentage of women (0.29%) opt for home births, often seeking a more personalized and natural birthing experience. Certified midwives support homebirth in Greece, but private arrangements, rather than those of the Ministry of Health and Primary Healthcare, primarily facilitate it. Midwifery-led care is available and increasingly recognized for its benefits, especially within hospital settings and private birthing centers^11-15^.

Given the increasing emphasis on patient-centered care and shared decision-making in maternal healthcare, the availability of a culturally adapted and validated instrument like the MADM scale is paramount for promoting respectful and empowering care practices in Greece.

The primary objective of this study is to validate the MADM scale in women who had at least one home childbirth experience in Greece. Specifically, the study aims to: 1) assess the psychometric properties of the MADM scale, including its reliability and validity, within the Greek cultural context; 2) evaluate the cultural appropriateness and linguistic clarity of the MADM scale items for women; 3) examine the associations between maternal autonomy measured by the validated MADM scale and various sociodemographic and clinical factors; and 4) explore the implications of maternal autonomy in decision-making for maternal healthcare practices and outcomes in Greece.

## METHODS

### Study design

We conducted a retrospective online survey among Greek women with at least one home childbirth experience from January 2010 to December 2023 using an online self-administered questionnaire. The study was limited to those giving birth at home because the aim was to validate the Mother’s Autonomy in Decision Making (MADM) scale within a specific context that differs significantly from hospital births. Home births often involve different levels of autonomy, decision-making processes, and interactions with healthcare providers compared to hospital births. By focusing on women who had experienced home childbirth, the study could more accurately assess the reliability and validity of the MADM scale in evaluating maternal autonomy in this particular setting, where women may have more control over their birth plans and fewer medical interventions. This context-specific validation ensures the scale’s applicability and relevance to the unique experiences of home childbirth in Greece.

### Population studied

The inclusion criteria were: 1) age >18 years; 2) agreement to participate in the study; and 3) experienced their last pregnancy within ten years of data collection. Responses about childbirth experiences from outside Greece were excluded from the dataset.

### Exclusion criteria

The exclusion criteria were: 1) incomplete survey responses (participants who did not complete the entire questionnaire); and 2) participants who refused to provide informed consent. To maintain data integrity and reliability, only complete survey responses were included in the final analysis. Participants who began the survey but did not complete it were excluded from the dataset. This approach ensured that all analyzed data met the study’s standards for completeness and accuracy.

### Questionnaire used

We used data from the literature to create a self-administered questionnaire with 11 questions (Supplementary file). We divided the questionnaire into three sections: 1) demographics of the study population (Q 1–6); 2) data regarding preparation for home birth, data regarding income and profession before home birth, and data on which professionals attended the home birth (Q 7–10); and 3) knowledge about childbirth rights and laws (Q 11). We also used the Greek version of the MADM scale (Supplementary file).

### MADM scale

We first obtained the necessary permission to convert the MADM scale to Greek by contacting the scale developers via email. The scale was translated into Greek by an expert who was fluent in both languages. The translated scale was evaluated by experts, and corrections were made in line with their opinions. The translated scale was then translated back into English by individuals who were proficient in the language.

MADM scale consists of 7 items and is a 6-point Likert-type scale, rated from 1 = ‘strongly disagree’ to 6 = ‘strongly agree’. The scale score range is 7–42, and the higher the scores, the higher the ability to lead decisions about care. Scores of 7–15 are reported as ‘very low autonomy’, 16–24 as ‘low autonomy’, 25–33 as ‘moderate level autonomy’, and 34–42 as ‘high autonomy’^4^.

### Data collection

We used the following online recruitment strategies to reach the targeted participants: 1) Online Survey Platform: the Microsoft Forms electronic platform hosted a self-administered questionnaire that collected data. We selected this platform due to its user-friendly interface and accessibility; 2) Social Media Outreach: we disseminated the URL link to the online questionnaire via social media, specifically through closed Facebook groups. These groups included communities of women who had given birth at home, thus directly targeting our population of interest; 3) Infographic Promotion: we electronically shared a specially designed infographic that highlighted the study’s purpose and the importance of participation. We sent this infographic to various women’s groups, midwifery organizations, and associations known to have members who had experienced home childbirth; and 4) Midwifery Networks: collaboration with midwifery organizations and professionals who provided care for home births was crucial. These professionals helped spread the word about the study within their networks, further ensuring that the survey reached potential participants.

### Ethical considerations

Participation in the survey was voluntary. We provided participants with a concise paragraph explaining the study’s objectives and assuring them of the confidentiality of their responses before starting the questionnaire. We obtained informed consent from all participants. The data focused on the first home birth. The study protocol was approved by the Research Ethics Boards of the University of West Attica (protocol number 77034/01-09-2023).

### Statistical analysis

Using the Kolmogorov-Smirnov test, the distributions of the continuous variables were tested for normality. Mean values and standard deviations (SDs) were used to describe those that were normally distributed, while medians and ranges were additionally used for those that were not normally distributed. Absolute (n) and relative (%) frequencies were used to describe categorical variables. The non-parametric Mann-Whitney U and the Kruskal-Wallis tests were used to compare non-normally distributed variables among different categories. Spearman’s correlation coefficient was used to test the relationship between two continuous variables.

We used the confirmatory factor analysis with a maximum likelihood procedure to assess the construct validity and confirm the factors of the MADM scale for mothers’ autonomy.

Several approaches were used to assess the fit of confirmatory factor analysis models, including comparative fit index (CFI), goodness-of-fit indices, Tucker-Lewis index (TLI), and root mean square error of approximation (RMSEA). The CFI and TLI can take values from 0 to 1, and a good fit to the data is considered when it is close to or above 0.9 or with even stricter criteria when it is close to or above 0.95. RMSEA values less than 0.05 indicate a good fit, and values up to 0.08 indicate an acceptable fit. We used Cronbach’s α to assess the internal consistency of the scale, ensuring that the items within the scale are consistently measuring the same construct. Significance levels are two-sided, and statistical significance was set at 0.05. The statistical program SPSS 26.0 was used for the analysis.

## RESULTS

### Demographics

The sample consists of 162 women with a mean age of 36.4 years (SD=5.4 years); 94.4% of the participants had Greek nationality, and 54% lived in Attica. Also, 50.6% had a Bachelor’s degree, 82.1% were married, and 46.9% had two children. Table 1 summarizes the demographic characteristics of the study population.

**Table 1:** Demographic characteristics of the study population

*Characteristics*		*n*	*%*
**Age** (years), mean (SD)		36.4 (5.4)	
**Nationality**	Other	9	5.6
	Greek	153	94.4
**Prefecture of residence: Attica**	No	74	46
	Yes	87	54
**Education level**	No education	1	0.6
	High school	7	4.3
	Technical school	3	1.9
	Vocational school for two years of training	13	8
	Bachelor’s degree	95	50.6
	Master’s degree	51	31.5
	Doctorate	5	3.1
**Marital status**	Married	133	82.1
	Single	5	3.1
	Divorced	4	2.5
	Cohabitation agreement	15	9.3
	Cohabitation	5	3.1
**Number of children**	1	28	17.3
	2	76	46.9
	3	42	25.9
	4	8	4.9
	5	5	3.1
	6	3	1.9

### Preparation for home birth, data regarding income, profession before home birth, and data on which professionals attended the home birth

Most participating women (71%) attended antenatal preparation classes from a midwife before delivery. Most of them (46.3%) had a family income of 1000–2000 euros per month, and most of them (34.2%) were private employees; 21.7% were self-employed, 13.7% unemployed, and 10.6% civil servants.

Among the participants, 73.5% had two midwives during the home birth, and 13% also had a doula. For 93.2% of the participants, there was at least one midwife during the delivery, while for 4.3% there was no midwife.

Data regarding preparation for home birth, income, profession before home birth, and which professionals attended the home birth, are given in Table 2.

**Table 2:** Data regarding preparation for home birth, data regarding income and profession before home birth, and data on which professionals attended the home birth

*Question*		*n*	*%*
**In your first home birth, did you attend antenatal preparation courses with a midwife?**	No	38	23.5
	Yes	115	71
	I am a midwife	9	5.6
**Your monthly household income during your first home birth was?** (€)	500–1000	46	28.4
	1000–2000	75	46.3
	2000–3000	26	16
	3000–4000	8	4.9
	>4000	7	4.3
**What was your job when you had your first home birth?**	Private employee	55	34.2
	Civil servant	17	10.6
	Self-employed	35	21.7
	Healthcare professional	8	4.9
	Midwife	9	5
	Unemployed	22	13.7
	Householder	16	9.9
***Question***		***n***	***%***
**Which professionals attended your first home birth?**			
2 midwives		119	73.5
1 midwife		17	10.3
1 midwife and 1 doula		11	6.8
1 midwife and 1 gynecologist		5	3.1
1 doula		21	13
Gynecologist		6	3.7
Pediatrician		5	3.1
Acupuncturist		3	1.9
Reflexologist		2	1.2
Osteopath		1	0.6
Unassisted (absence of health care professional)		4	2.5
Other		10	6.2

### Knowledge about childbirth rights and laws

Among the participants, 32.1% were very/extremely aware of hospitalized patient’s rights, 39.5% of current home birth laws, 43.8% of children’s rights, and 48.8% of sexual and reproductive rights. Table 3 shows the data regarding knowledge about childbirth rights and laws.

**Table 3:** Knowledge about childbirth rights and laws

*To what extent do you know*		*n*	*%*	*Very well/extremely well %*
**The rights of a hospitalized patient?**	Not at all	16	9.9	32.1
	Slightly	56	34.6	
	Moderately	38	23.5	
	Very well	22	13.6	
	Extremely well	30	18.5	
**The current home birth laws?**	Not at all	7	4.3	39.5
	Slightly	42	25.9	
	Moderately	49	30.2	
	Very well	31	19.1	
	Extremely well	33	20.4	
**The children’s rights?**	Not at all	8	4.9	43.8
	Slightly	30	18.5	
	Moderately	53	32.7	
	Very well	36	22.2	
	Extremely well	35	21.6	
**Your sexual and reproductive rights?**	Not at all	10	6.2	48.8
	Slightly	25	15.4	
	Moderately	48	29.6	
	Very well	38	23.5	
	Extremely well	41	25.3	

### MADM scale

The MADM scale ranged from 16 to 42 points with a median value of 38.

Table 4 illustrates MADM scale item score percentages according to participants’ answers.

**Table 4:** MADM scale item score percentages according to participants’ answers

	*Strongly disagree %*	*Disagree %*	*Somewhat disagree %*	*Somewhat agree %*	*Agree %*	*Strongly agree %*
My doctor or midwife asked me how involved in the decision making I wanted to be	3.7	1.9	6.2	15.4	32.1	40.7
My doctor or midwife told me that there are different options for my maternity care	1.9	1.2	1.9	15.4	31.5	48.1
My doctor or midwife explained the advantages/disadvantages of the maternity care options	1.2	1.2	2.5	16.7	33.3	45.1
My doctor or midwife helped me understand all the information	1.2	0.6	1.9	11.7	31.5	53.1
I was given enough time to thoroughly consider the different care options	0	1.2	1.9	9.9	33.7	53.7
I was able to choose what I considered to be the best care options	0	0	0.6	7.4	25.9	66
My doctor or midwife respected my choices	1.9	0	0.6	8	21.6	67.9

### Confirmatory factor analysis

Confirmatory factor analysis revealed an acceptable fit for the questionnaire, where the CFI and TLI indices were greater than 0.9 (0.92 and 0.91, respectively), and the RMSEA index was acceptable and equal to 0.07. The correlation coefficients of each question with the overall respect dimension in decision-making regarding pregnancy or childbirth care were acceptable. Also, it would not improve the reliability factor if any of the questions were removed, so all questions remain within the factor. Cronbach’s α reliability coefficient was 0.92 (greater than 0.7), indicating acceptable reliability. Table 5 presents the correlations of the questions and the Cronbach’s reliability coefficient.

**Table 5:** Correlations of the questions and Cronbach’s reliability coefficient

	*Corrected item-total correlation*	*Cronbach’s α if item deleted*	*Cronbach’s α*
MADM-1	0.652	0.920	0.92
MADM-2	0.836	0.892	
MADM-3	0.825	0.893	
MADM-4	0.874	0.889	
MADM-5	0.757	0.902	
MADM-6	0.642	0.914	
MADM-7	0.707	0.906	

MADM: Mother’s Autonomy in Decision Making.

### Association of MADM scale score with participants’ characteristics

**Figure 1:**
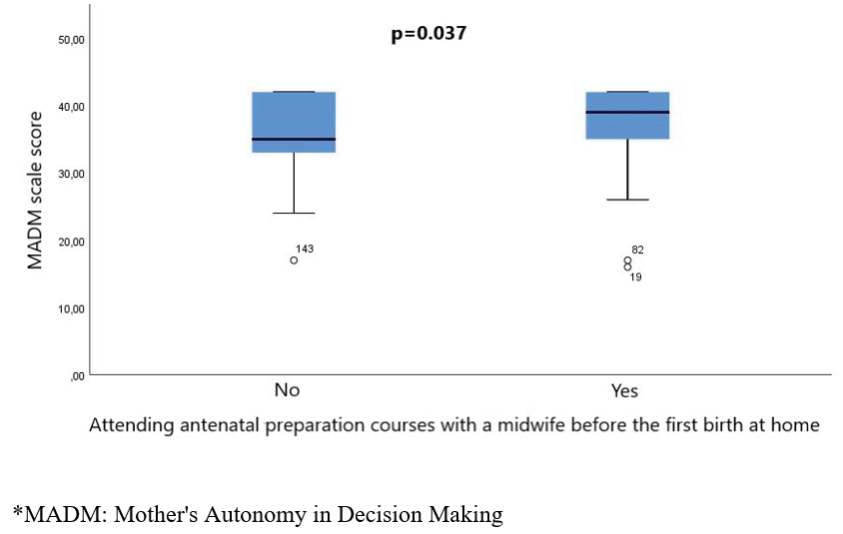
Median values of MADM scale score in the women who had attended antenatal preparation courses with a midwife before their first home birth compared to those who had not

We detected a weak negative statistically significant correlation between the age of the participants and the MADM scale score (Spearman’s rho= -0.166, p=0.035). We also found a statistically significantly higher median value of MADM scale score in the women who had attended antenatal preparation courses with a midwife before their first home birth, compared to those who had not [MADM scale score 39 (range: 17–42) vs 35 (range: 16–42), p=0.037] (Figure 1).

## DISCUSSION

The utilization of the MADM scale provided valuable insights into participants’ autonomy in prenatal care and childbirth decision-making processes. The scale demonstrated good reliability and validity, indicating its utility as a tool for assessing maternal autonomy in the Greek context. This finding is consistent with earlier studies that underscored the favorable reliability and validity of the MADM scale across different nations^5-9^.

Previous research has mentioned the influence of age on women’s empowerment and decision-making autonomy. Acharya et al.^16^ observed that increased age is positively associated with women’s autonomy in decision-making.

In our study, the negative correlation between age and MADM scale scores suggests that younger women may exhibit greater autonomy in decision-making regarding pregnancy and childbirth. This could be explained by the fact that in developed countries like Greece, younger women may be more influenced by changing societal norms and attitudes towards autonomy in decision-making regarding pregnancy and childbirth. Younger generations may be more inclined to assert their autonomy and independence compared to older generations, who may adhere more closely to traditional norms. With the proliferation of information through the internet and social media, younger women may have greater access to resources and support networks that provide information on reproductive health and rights. Having access to information empowers individuals to make informed decisions and advocate for their preferences throughout the stages of pregnancy and childbirth^17^.

Furthermore, there was an association between attending antenatal preparation courses with a midwife and higher MADM scores. This suggests that education and information provision, as facilitated by midwives, may be related to greater maternal autonomy. However, due to the cross-sectional nature of the study, it is not possible to determine whether attending these courses led to higher autonomy scores, whether women with higher autonomy were more likely to attend such courses, or if another variable influenced both. Several studies have reported associations between childbirth education and increased confidence in decision-making among pregnant women^18,19^. Strengthening educational initiatives and promoting access to evidence-based information may enhance women’s autonomy and decision-making capabilities throughout the perinatal period. Moreover, the involvement of midwives during home births was widespread among participants, highlighting the pivotal role of midwifery care in supporting women throughout the childbirth process. Midwifery-led care models globally enhance women’s decision-making and autonomy during childbirth by providing continuous, personalized support and empowering women to make informed choices about their care. Studies have shown that these models not only improve maternal satisfaction and health outcomes but also respect and uphold the autonomy of women, particularly in settings that emphasize patientcentered care^20-23^.

In addition, the findings of this study shed light on various aspects of maternal healthcare among Greek women with home childbirth experience. The demographic profile of the participants reveals key characteristics that may influence maternal health outcomes and decision-making processes. The majority of the participants were well-educated, married, and had two children. This demographic profile aligns with previous research indicating that education level and marital status can impact maternal healthcare choices and outcomes^11,24^.

Another interesting finding of the study is that participants exhibited varying levels of awareness regarding childbirth rights and laws, with notable gaps in knowledge observed. While the study did not find a direct association between knowledge of rights and MADM scores, improving women’s understanding of their rights and legal protections during pregnancy and childbirth, remains important for advocating for respectful maternity care^25^.

### Strengths and limitations

The study addresses a significant concern in maternal healthcare, emphasizing the importance of informed decision-making for expectant mothers, which is crucial for maternal and neonatal health outcomes. It contributes to the validation of the MADM scale in a specific context – women who have experienced home childbirth in Greece. This adds to the broader understanding of the scale’s applicability across different cultural and healthcare settings. The study includes a reasonable sample size of 162 women, providing insights into the autonomy of women who opt for home childbirth in Greece. The predominantly Greek sample enhances the study’s relevance to the local context. The use of confirmatory factor analysis to assess the psychometric properties of the MADM scale enhances the study’s methodological rigor. The reported fit indices (CFI, TLI, and RMSEA) and internal consistency (Cronbach’s α) demonstrate the reliability and validity of the scale in the studied population. In addition, the study explores associations between demographic factors (age) and maternal autonomy, as well as the impact of attending antenatal preparation courses with a midwife on autonomy scores. These findings provide valuable insights into potential factors associated with maternal decision-making autonomy.

The study has some limitations. The study relies on data collected through an online survey, which may introduce sampling bias as it only includes women who have internet access and are willing to participate in online research. This could limit the generalizability of the findings to the broader population of women who opt for home childbirth in Greece. The retrospective nature of the study introduces the possibility of recall bias, as participants are asked to recollect past experiences and decisions related to childbirth, which may affect the accuracy of responses. While the study provides insights into maternal autonomy, specifically within the context of home childbirth in Greece, the findings may not be generalizable to women with different cultural backgrounds or those opting for hospital births. Also, the current research identifies associations between age, attendance at antenatal preparation courses with a midwife, and MADM scores; however, there may be confounding variables not accounted for in the analysis that could influence these relationships. Moreover, the design of the study limits the ability to establish causality between attending midwifery courses and higher MADM scores or to determine the directionality of the observed associations.

## CONCLUSIONS

The study findings highlight significant aspects of maternal healthcare among Greek women with home childbirth experience. Most participants were well-educated, married, and had two children. Antenatal preparation courses with a midwife were common, and midwives played a central role during home births. The MADM scale demonstrated good reliability and validity. Age was negatively correlated with MADM scale scores. Notably, attending antenatal preparation courses with a midwife was associated with higher MADM scores, underscoring the importance of education for maternal autonomy. This study is novel in its application of the MADM scale to a Greek population, specifically focusing on home childbirth – a setting that inherently offers higher autonomy compared to hospital births. The validation of the MADM scale in this context contributes to the global understanding of maternal autonomy, providing a reliable tool for further research and practice in Greece and similar cultural settings.

## Supplementary Material



## Data Availability

The data supporting this research are available from the authors on reasonable request.
