# Supplementary material for: Validation of the Greek version of Mother's Autonomy in Decision Making (MADM) scale
Source: Eur J Midwifery. 2024 Jul 8;8:10.18332/ejm/189495. doi: 10.18332/ejm/189495 (PMC11229043; doi:10.18332/ejm/189495)
Supplement: Supplementary file 1 [file EJM-8-40-s1.pdf]

QUESTIONNAIRE

SECTION 1: Demographics

1. Age (Years):

2. Nationality

1. Greek

2. Other

3. Prefecture of residence

1. Attica

2. Thessaloniki

3. Evoia

4. Voiotia

5. Achaia

6. Other

4. Educational status

1. No education

2. Primary school

3. High school

4. Technical school

5. Vocational school for two years of training

6. Bachelor’s degree

7. Master’s degree

8. Doctorate

5. Marital status

1. Married

2. Single

3. Divorced

4. Widowed

5. Cohabitation agreement

6. Cohabitation

7. Other

6. Number of children:

SECTION 2: Data regarding preparation for home birth, data regarding income and profession before it and data on which professionals attended the home birth

7. In your FIRST home birth, did you attend antenatal preparation courses with a midwife?

1. Yes

2. No

3. I am a midwife

8. Your monthly household income during your FIRST home birth was:

1. 500-1000€

2. 1000-2000€

3. 2000-3000€

4. 3000-4000€

5. >4000€

9. What was your job when you had your FIRST home birth?

1. Private employee
2. Civil servant
3. Self-employed
4. Retired
5. Health care professional
6. Midwife
7. Unemployed
8. Householder
9. Other

10. Which professionals attended your FIRST home birth?

1. 2 midwives
2. 1 midwife
3. 1 midwife and 1 doula
4. 1 midwife and 1 gynecologist
5. 1 doula
6. Gynecologist
7. Pediatrician
8. Acupuncturist
9. Reflexologist

10. Osteopath
11. Unassisted (absence of health care professional)
12. Other

SECTION 3: Knowledge about childbirth rights and laws

11. In what extent do you know about:

	Not at all	Slightly	Moderately	Very well	Extremely well
**The rights of hospitalized patient?**					
**The current home birth laws?**					
**The children's rights?**					
**Your sexual and reproductive rights?**					

Η ΚΛΙΜΑΚΑ MADM ΠΕΡΙ ΤΗΣ ΑΥΤΟΝΟΜΙΑΣ ΤΩΝ ΜΗΤΕΡΩΝ

ΣΤΗ ΛΗΨΗ ΑΠΟΦΑΣΕΩΝ

ΟΔΗΓΙΕΣ

Κλίμακα MADM περί της αυτονομίας των μητέρων στη λήψη αποφάσεων

Η κλίμακα MADM περί της αυτονομίας των μητέρων στη λήψη αποφάσεων αναπτύχθηκε από γυναίκες για να περιγράψουν τις εμπειρίες τους από τη μαιευτική φροντίδα που είχαν. Η κλίμακα MADM είναι ένα αξιόπιστο και έγκυρο εργαλείο που αξιολογεί το επίπεδο αντιπροσώπευσης και αυτονομίας που νιώθει ένα άτομο όταν συμμετέχει σε συνομιλίες λήψης αποφάσεων που αφορούν την υγεία του με έναν πάροχο υγείας (γιατρό ή μαία) (Vedam el al, 2017).

Το εργαλείο, το σύστημα βαθμολόγησης και οι οδηγίες χρήσης παρουσιάζονται σε αυτό το έγγραφο. Η κλίμακα είναι επίσης διαθέσιμη για λήψη σε πολλές γλώσσες (βλ. Ισπανικά παρακάτω). Επικοινωνήστε μαζί μας εάν αναζητάτε έγκυρες μεταφράσεις σε άλλες γλώσσες.

Για περισσότερες πληροφορίες σχετικά με την ανάπτυξη της κλίμακας MADM διαβάστε τη δημοσίευση του 2017 εδώ.

Αυτή η κλίμακα είναι δωρεάν για χρήση σε ακαδημαϊκά περιβάλλοντα, κλινικά γραφεία ή ιδρύματα υγειονομικής περίθαλψης. Για ακαδημαϊκές σπουδές και ιδρύματα υγείας, δεν υπάρχει χρέωση, αλλά απαιτείται Συμφωνία Χρήστη για κάθε μελέτη και κάθε χρήση. Κάντε κλικ εδώ για να συμπληρώσετε τη φόρμα αιτήματος λήψης.

zxereatlve �commons This work is licensed under the Creative Commons Auribuuon 4.0 International License. To view a copy ofthis license, visit hup://creativecommons.org/licenses/by/4.0/ or send a letter to Creative Commons, PO Box 1866, Mountain View, CA 94042, USA

Ωστόσο, για οποιαδήποτε μελέτη που διεξάγεται από εμπορικό όμιλο ή εάν η μελέτη είναι, εν όλω ή εν μέρει, με την υποστήριξη ενός εμπορικού ή κυβερνητικού οργανισμού, μπορεί να καταβληθεί ένα τέλος. Αυτή η κλίμακα αμοιβής εξαρτάται από διάφορους παράγοντες. Για πληροφορίες παρακαλούμε επικοινωνήστε με το The Birth Place Lab απευθείας: laura.beer@ubc.ca

Τυχόν κεφάλαια που προέρχονται μέσω της συμφωνίας πνευματικών δικαιωμάτων χρησιμοποιούνται αποκλειστικά για την υποστήριξη των συνεχιζόμενων εργασιών του The Birth Place Lab.

Επικοινωνήστε μαζί μας για περαιτέρω πληροφορίες: laura.beer@ubc.ca

MADM CITATION:

Vedam, S., Stoll, K., Martin, K., Rubashkin, N., Partridge, S., Thordarson, D., & Jolicoeur, G. (2017). The Mother's Autonomy in Decision Making (MADM) scale: Patient-led development and psychometric testing of a new instrument to evaluate experience of maternity care. Plos One,12(2). doi:10.1371/journal.pone.0171804
http://journals.plos.org/plosone/article?id=10.1371/journal.pone.0171804

This work is licensed under the Creative Commons Attribution 4.0 International License. To view a copy of this license, visit http://creativecommons.org/licenses/by/4.0/ or send a letter to Creative Commons, PO Box 1866, Mountain View, CA 94042, USA

Η ΑΥΤΟΝΟΜΙΑ ΤΩΝ ΜΗΤΕΡΩΝ ΣΤΗ ΛΗΨΗ ΑΠΟΦΑΣΕΩΝ: Η ΚΛΙΜΑΚΑ MADM

Παρακαλούμε πείτε μας για τις συζητήσεις με το γιατρό ή τη μαία σας σχετικά με τις επιλογές της φροντίδα σας (για παράδειγμα: τον προγεννητικό σας έλεγχο, την έναρξη του τοκετού, τα φάρμακα, το πού και πώς θα γεννήσετε, τη φροντίδα του νεογνού, το αν θα πρέπει ή όχι να κάνετε καισαρική τομή, κλπ.).

Οι απαντήσεις μου περιγράφουν τις συζητήσεις μου ή τις εμπειρίες μου με:

Τον οικογενειακό γιατρό Το γυναικολόγο/Μαιευτήρα Τη μαία Δεν έχω κάποιον συγκεκριμένο από όλους προαναφέρθηκαν (πάω στο νοσοκομείο / Κέντρο Υγείας / ΙΚΑ της περιοχής μου)

Παρακαλώ περιγράψτε την εμπειρία σας σχετικά με τη λήψη αποφάσεων στη διάρκεια της εγκυμοσύνης σας και του τοκετού σας (της γέννας σας) (επιλέξτε μία επιλογή για το κάθε ένα)

	**Διαφωνώ απόλυτα**	**Διαφωνώ**	**Εν μέρει διαφωνώ**	**Εν μέρει συμφωνώ**	**Συμφωνώ**	**Συμφωνώ απόλυτα**
Ο γιατρός ή η μαία μου με ρώτησε πόσο θέλω να συμμετέχω στη λήψη αποφάσεων	1	2	3	4	5	6
Ο γιατρός ή η μαία μου με ενημέρωσαν ότι υπάρχουν διαφορετικές επιλογές στη μαιευτική φροντίδα	1	2	3	4	5	6
Ο γιατρός ή η μαία μου μου εξήγησαν τα οφέλη και τα μειονεκτήματα κάθε μίας διαφορετικής επιλογής στη μαιευτική φροντίδα	1	2	3	4	5	6
Ο γιατρός ή η μαία μου με βοήθησαν να καταλάβω όλες τις πληροφορίες	1	2	3	4	5	6
Μου δόθηκε αρκετός χρόνος να σκεφτώ διεξοδικά τις διαφορετικές επιλογές που είχα στη φροντίδα μου	1	2	3	4	5	6
Μπόρεσα να επιλέξω αυτό που εγώ θεώρησα την καλύτερη επιλογή	1	2	3	4	5	6
Ο γιατρός ή η μαία μου σεβάστηκαν τις επιλογές μου	1	2	3	4	5	6
	**ΣΥΝΟΛΙΚΟ ΑΡΘΡΟΙΣΜΑ / ΒΑΘΜΟΛΟΓΙΑ:**					

zxereatlve �commons This work is licensed under the Creative Commons Auribuuon 4.0 International License. To view a copy ofthis license, visit hup://creativecommons.org/licenses/by/4.0/ or send a letter to Creative Commons, PO Box 1866, Mountain View, CA 94042, USA

Το εύρος της βαθμολογίας είναι 7-42, με το υψηλότερο σκορ να δείχνει περισσότερες ευκαιρίες για να αναλάβουν οι γυναίκες ενεργό ρόλο και να καθοδηγούν τις αποφάσεις.

**ΚΛΕΙΔΙΑ<br>Επίπεδο Αυτονομίας<br>(Ανά τεταρτημόρια)**	
**Συνολική Βαθμολογία**	**Ένδειξη Σεβασμού**
7-15	Πολύ χαμηλή αυτονομία ασθενούς - επιτόκου
16-24	Χαμηλή αυτονομία ασθενούς – επιτόκου
25-33	Μέτρια αυτονομία ασθενούς - επιτόκου
34-42	Υψηλή αυτονομία ασθενούς - επιτόκου

This work is licensed under the Creative Commons Attribution 4.0 International License. To view a copy of this license, visit http://creativecommons.org/licenses/by/4.0/ or send a letter to Creative Commons, PO Box 1866, Mountain View, CA 94042, USA

© 2024 Serpetini E. et al.
